# The impact of the COVID-19 pandemic on people who use drugs in three Canadian cities: a cross-sectional analysis

**DOI:** 10.1186/s12954-024-00996-x

**Published:** 2024-05-16

**Authors:** Sanjana Mitra, Zachary Bouck, Sarah Larney, Camille Zolopa, Stine Høj, Nanor Minoyan, Katie Upham, Indhu Rammohan, Wing Yin Mok, Kanna Hayashi, M-J Milloy, Kora DeBeck, Ayden Scheim, Dan Werb

**Affiliations:** 1 Centre on Drug Policy Evaluation, St. Michael’s Hospital, Unity Health Toronto, 209 Victoria St, Toronto, ON M5B 1T8 Canada; 2 Department of Medicine, University of California, San Diego, USA; 3 Centre de Recherche du Centre Hospitalier de l’Université de Montréal, Montréal, Canada; 4 Département de Médecine Famille et de Médicine d’Urgence, Université de Montréal, Montréal, Canada; 5 Department of Educational and Counselling Psychology, McGill University, Montréal, Canada; 6 Department of Social and Preventive Medicine, École de Santé Publique, Université de Montréal, Montréal, Canada; 7 British Columbia Centre on Substance Use, Vancouver, Canada; 8 Faculty of Health Sciences, Simon Fraser University, Burnaby, Canada; 9 Division of Social Medicine, Department of Medicine, University of British Columbia, Vancouver, Canada; 10 School of Public Policy, Simon Fraser University, Burnaby, Canada; 11 Dornsife School of Public Health, Drexel University, Philadelphia, USA; 12 Department of Epidemiology and Biostatistics, Schulich School of Medicine and Dentistry, Western University, London, Canada; 13 Institute for Health Policy, Management and Evaluation, University of Toronto, Toronto, Canada; 14 Division of Infectious Diseases and Global Public Health, University of California San Diego, San Diego, United States

**Keywords:** People who use drugs, COVID-19, Pandemic, Social inequities, Emergency response

## Abstract

**Background:**

The COVID-19 pandemic had a disproportionate impact on the health and wellbeing of people who use drugs (PWUD) in Canada. However less is known about jurisdictional commonalities and differences in COVID-19 exposure and impacts of pandemic-related restrictions on competing health and social risks among PWUD living in large urban centres.

**Methods:**

Between May 2020 and March 2021, leveraging infrastructure from ongoing cohorts of PWUD, we surveyed 1,025 participants from Vancouver (*n* = 640), Toronto (*n* = 158), and Montreal (*n* = 227), Canada to describe the impacts of pandemic-related restrictions on basic, health, and harm reduction needs.

**Results:**

Among participants, awareness of COVID-19 protective measures was high; however, between 10 and 24% of participants in each city-specific sample reported being unable to self-isolate. Overall, 3–19% of participants reported experiencing homelessness after the onset of the pandemic, while 20–41% reported that they went hungry more often than usual. Furthermore, 8–33% of participants reported experiencing an overdose during the pandemic, though most indicated no change in overdose frequency compared the pre-pandemic period. Most participants receiving opioid agonist therapy in the past six months reported treatment continuity during the pandemic (87–93%), however, 32% and 22% of participants in Toronto and Montreal reported missing doses due to service disruptions. There were some reports of difficulty accessing supervised consumption sites in all three sites, and drug checking services in Vancouver.

**Conclusion:**

Findings suggest PWUD in Canada experienced difficulties meeting essential needs and accessing some harm reduction services during the COVID-19 pandemic. These findings can inform preparedness planning for future public health emergencies.

## Background

Beginning in 2020, the COVID-19 pandemic prompted unprecedented public health measures in Canada, including the declaration of provincial health emergencies; bans on public and private social gatherings; the closure of schools and non-essential businesses; the promotion of physical distancing to limit contact between individuals; and restrictions on the operation of some essential health and social services [1–4]. Evidence from previous “Big Events” (e.g., natural disasters, terrorist attacks, economic crises) demonstrates that equity-deserving groups, including people who use drugs, are often disproportionately impacted by such large-scale events, as disruptions to health and social services [5], intensified psychological distress [6], and financial precarity [7] jeopardize their ability to meet basic survival needs and negotiate health-related risks [8].

Canada and the United States have witnessed escalating rates of drug poisoning driven by the contamination of the unregulated drug supply since approximately 2016 [9]. In the year following the declaration of the COVID-19 public health emergency by the World Health Organization [10], fatal and non-fatal drug poisonings increased dramatically in Canada. For example, in the first 15 weeks of the COVID-19 emergency in Ontario, the weekly rate of opioid-related deaths increased 38% compared to the 15 weeks immediately preceding the pandemic [11]. In British Columbia, overdose deaths more than doubled in the nine months after the declaration of the provincial public health emergency compared to the nine months prior [12], while Quebec experienced a 28% increase in overdose mortality in the three months after COVID-19 restrictions were imposed compared to the three months prior [13]. Several investigations from across Canada and the US indicate that rising mortality is in part attributable to pandemic-related increases in drug use frequency and quantity, social isolation and using alone more often, changes in the drug supply, and reductions in access to harm reduction and drug treatment services [14–16]. This increase in drug-related fatalities suggests a trend reversal in the drug poisoning rate—which had begun to slow and even decline in 2019 in some Canadian settings—prior to the implementation of pandemic-related restrictions [17–19].

There has been an acceleration of viral spillover events and ensuing zoonotic epidemics over the past twenty years, and it is likely that future public health emergencies will emerge [20]. If we are to establish robust and equitable preparedness strategies prior to and during such emergencies it is imperative to examine the varied ways in which the COVID-19 pandemic and associated public health responses may have contributed to the exacerbation of drug poisoning deaths, and understand the influence of measures that sought to mitigate impacts on people who use drugs (e.g., makeshift temporary shelters, relaxed eligibility criteria for take-home doses of opioid agonist therapies). While some Canadian studies have examined the health-related and behavioural impact of the COVID-19 pandemic on people who use drugs [16, 21–24], few studies have compared the experiences people who use drugs across jurisdictions [16, 25]. In the Canadian context, the administration and provision of health services–including harm reduction, treatment, and wrapround supports for people who use drugs–fall under the primary responsibility of provinces and territories [26]. However, differences in resource allocation and policies have resulted in variation of support and implementation of these programs and services across settings [26]. We therefore undertook a rapid quantitative cross-sectional assessment of the impacts of COVID-19 on people who use drugs in Canada’s three largest cities: Vancouver, British Columbia; Toronto, Ontario; and Montreal, Quebec. We sought specifically to document commonalities and differences in potential sources of COVID-19 exposure and the impacts of pandemic-related restrictions on competing health and social risks among people who use drugs living in these urban centres.

## Methods

### Setting

#### Vancouver

On March 17th, 2020, the Provincial Health Officer declared a provincial state of emergency. The following day, the Government of British Columbia implemented associated measures through to mid-May 2020 as part of the province’s initial pandemic response, which included closures of non-essential services [4]. From mid-May 2020 onward, the province relaxed some of these restrictions and allowed for the reopening of more businesses and services with enhanced safety protocols [27]. Subsequently in June 2021, British Columbia initiated a four-step plan to end the provincial state of emergency [28]. The City of Vancouver implemented a number of measures to deliver essential services to its residents, including emergency response centres for people experiencing homelessness, increased provision of hygiene services and supplies, enhanced access to basic needs services, and safer options for Income Assistance disbursement [29]. Harm reduction and drug treatment services continued operation across the city, with capacity restrictions or adaptations to comply with public health guidelines for physical distancing [21].

#### Toronto

On March 17th, 2020, the Government of Ontario declared a state of emergency and implemented associated measures to close non-essential businesses, restrict social gatherings, and promote physical distancing [30]. In response to the pandemic, the City of Toronto opened COVID-19 Isolation and Recovery sites to provide safe isolation spaces for people experiencing instability [31]. Health service modifications due to physical distancing requirements limited the capacity of clinicians and health service providers to deliver care for people who use drugs [16]. Although most harm reduction services adapted to evolving public health requirements by reducing physical capacity of services and implementing screening requirements, the city’s busiest supervised consumption site reopened after a month-long closure after the initial declaration of the public health emergency [32]. Following the province-wide declaration, overall capacity restrictions and temporary closures at supervised consumption sites resulted in reductions of 25-50% in service user volume across the city [33].

#### Montreal

A provincial public health emergency was declared by the Government of Quebec on March 13th, 2020, closing schools, banning indoor assemblies of more than 250 people, and recommending self-isolation for 14 days for people recently returned from abroad [3]. Closure of all but essential businesses and services followed shortly after, as well as travel restrictions within the province [34]. The City of Montreal implemented a number of crisis responses, including outdoor day centres in parks to offer meals and sanitary facilities, improvised shelters in arenas and hotels, and a screening and isolation unit at the former Royal-Victoria Hospital [35]. A number of social services including day centres and shelters closed or significantly limited their operations. Three of the city’s four supervised consumption sites closed temporarily in the weeks following the declaration of the health emergency due to lack of resources to comply with public health measures, but progressively re-opened beginning in mid-May 2020 with access restrictions in place. Other harm reduction services operated at reduced capacity and limited hours in the weeks and months following the initial lockdown phase [23].

### Participants

Participants were almost exclusively sampled from ongoing cohort studies of people who use drugs surveyed during the COVID-19 pandemic.

#### Vancouver

Participants were drawn from three longstanding community-recruited prospective cohorts of people who use drugs living in Vancouver, including: (1) the Vancouver Injection Drug Users Study (VIDUS; began enrollment in 1996), which consists of HIV-negative adults who inject drugs [36]; (2) the AIDS Care Cohort to evaluate Exposure to Survival Services (ACCESS; began enrollment in 2005 [37]), which consists of adults living with HIV who use drugs; and (3) the At-Risk Youth Study (ARYS; began enrollment in 2005), which consists of street-involved youth (aged 14–26 years at enrolment) who use drugs [38]. Participants are recruited through convenience-based sampling by a diverse range of methods including, passive recruitment (posters, cards), active street-based outreach, snow-ball sampling and word-of mouth, via health and social service agencies, single-occupancy hotels, and storefronts from across Vancouver’s downtown core [36, 37]. For the present analysis, the sample was restricted to those who reported having used any drugs in the past six months, excluding those who only used cannabis.

#### Toronto

Participants in Toronto were drawn from the prospective Ontario integrated Supervised Injection Services Toronto (OiSIS-Toronto) cohort, which consists of adults who inject drugs living in the city and began recruitment in November 2018 [39]. Participants of the OiSIS-Toronto cohort were recruited through convenience-based sampling (i.e., active outreach, on-site recruitment, and passive recruitment) from predominantly three supervised consumption sites established across the city [39]. For the present study, the sample was restricted to those reporting past six-month drug use, excluding those who only used cannabis.

#### Montreal

Montreal-based participants were drawn from two sources: (1) the Hepatitis Cohort (HEPCO), a community-based cohort of people who inject drugs in Montreal initiated in 2004 to investigate factors associated with incident HCV cases and the natural history of HCV infection [40]; and (2) a convenience-based sample of people who use drugs recruited from October to December 2020 via community-based organizations providing low-threshold services (e.g., day centres, harm reduction services, shelters) through posters and outreach visits from a community liaison with lived experience. Eligibility criteria for HEPCO includes past six-month drug use and being 18 years or older [23]. Individuals eligible for the latter sample included those aged 18 years or over and reporting past-year drug use [23]. Those reporting only cannabis use within the respective specified time frame for drug use were excluded from either sample.

### Data collection

A common set of questions to investigate the impact of COVID-19 on the health and wellbeing of people who use drugs in Canada was developed across all three sites. Questionnaire development was guided by a systematic review of health needs assessments in disaster contexts [41]. In brief, the survey items collected self-reported data from participants about their (1) awareness of, and capacity to adhere to COVID-19 public health guidelines; (2) COVID-19 concerns and experience of exposure and testing; as well as (3) the impact of COVID-19 on their ability to meet essential needs (e.g., housing, income, food security); (4) ability to access drug treatment and harm reduction services; and (5) frequency of overdose and associated drug use behaviours. Studies using measures derived from this set of COVID-19 related questionnaire items have been published elsewhere [22–24]. Unless stated otherwise, questions were phrased to assess changes in behaviour/circumstance since the declaration of the public health emergency in each respective setting. The comparison period was usually not defined, although most questions asked participants to compare their behaviour or circumstance during the COVID-19 pandemic to their experiences immediately prior.

In all settings, questionnaires were administered by interviewers trained in engaging with people with lived or living experience of substance use. Interviews took anywhere between 45 and 90 min to complete and participant responses were recorded via an electronic data capturing system or online survey platform. Data collection included both in-person (with physical distancing and personal protective equipment in use) and remote (e.g., telephone, videoconferencing) options with interviews conducted in compliance with public health and sanitary measures in effect at the time. A detailed description of COVID-19 site-specific data collection procedures can be found elsewhere [22, 24]. Given that COVID-19 restrictions were enacted and relaxed at varying times across the three cities, each site commenced data collection at different times. Vancouver participants were surveyed between July 2020 and November 2020; Toronto participants were surveyed between June 2020 and March 2021; and Montreal participants were surveyed between May 2020 and December 2020. All participants were compensated between $30 and $50 CAD depending on the site for completing their interview. Site-specific project protocols and questionnaires were reviewed and approved by the Providence Health Care/University of British Columbia Research Ethics Board in Vancouver; the Research Ethics Board of St. Michael’s Hospital (Unity Health Toronto) in Toronto; and the Research Ethics Committee of the Centre Hospitalier de l’Université de Montréal in Montreal.

### Analysis

Given differences in the underlying data sources (e.g., varying cohort eligibility criteria) for each city-specific sample as well as small cell sizes, we did not undertake formal statistical comparisons across sites. Instead, we report the frequency (number and proportion) of responses to common survey items to describe key pandemic-related health and social experiences among participants in each city during the relevant study period.

## Results

Overall, 1,025 participants were recruited across all three sites, including 640 (62%) in Vancouver, 158 (15%) in Toronto, and 227 (22%) in Montreal (129 from HEPCO and 98 from the service-based convenience sample). Demographic characteristics of participants from each site are presented in Table 1. Participants in each sample tended to be middle-aged, and a large proportion were cisgender men. A majority of participants across each sample were white (53-81%), although the Vancouver sample had a larger proportion of Indigenous participants (39%) compared to the other two sites (24% and 5% in Toronto and Montreal). Furthermore, 37-56% of participants reported current or past six-month experiences of unstable housing and 5-12% of participants reported past six-month incarceration.

**Table 1:** Sociodemographic characteristics of sampled people who use drugs during the COVID-19 pandemic across three Canadian cities, May 2020–March 2021 (*N* = 1,025)

Characteristic	Vancouver (*N* = 640)	Toronto (*N* = 158)	Montreal (*N* = 227)
**Gender** ^**1,2**^ **, n (%)**			
Cisgender men	355 (59)	106 (67)	174 (77)
Cisgender women	234 (39)	41 (26)	49 (22)
Transgender or other gender identity	13 (2)	9 (6)	4 (2)
**Age (median, IQR)** ^**3**^	45 (32–55)	42 (34–49)	47 (40–57)
**Ethnicity**^**4**^, **n (%)** Indigenous Non-Indigenous Person of Colour white	248 (39) 32 (5) 356 (56)	38 (24) 34 (22) 84 (53)	12 (5) 31 (14) 182 (81)
**Unstable housing status**^**5,6**^, **n (%)**	357 (56)	78 (49)	83 (37)
**Recent incarceration**^**7**^, **n (%)**	31 (5)	19 (12)	11 (5)

*Notes*: All percentages are calculated with missing responses included in the denominator.

IQR = interquartile range.

^1^Ascertained by asking participants to self-identify their gender.

^2^Excludes one refusal and one missing value in Toronto, and 38 missing values in Vancouver.

^3^Excludes one missing value in Montreal.

^4^Excludes two missing values in Montreal, two “don’t know” in Toronto, and four missing values in Vancouver.

^5^Reflects current unstable housing status in Vancouver, and past six-month unstable housing status in Toronto and Montreal.

^6^Excludes three missing values in Vancouver.

^7^Excludes seven missing values in Vancouver.

### COVID-19 concerns, exposure, and testing

Overall, 55% (*n* = 350) of Vancouver participants, 65% (*n* = 102) of Toronto participants, and 45% (*n* = 102) of Montreal participants reported having concerns about COVID-19. Of those who reported having concerns, the most common were getting sick (64% in Vancouver; 35% in Toronto; and 46% in Montreal), family and loved ones getting sick (31% in Vancouver; 19% in Toronto; and 21% in Montreal), and dying (19% in Vancouver; 8% in Toronto; and 6% in Montreal). In contrast, few participants were concerned about changes in access to drugs, withdrawal, limited access to harm reduction and treatment services, and ability to maintain physical distancing requirements. In Vancouver, 29% of participants had been tested for SARS-CoV-2; in Toronto, 64%; and in Montreal, 35%. Few of these participants tested positive for SARS-CoV-2: three participants in Vancouver, three participants in Toronto, and one participant in Montreal self-reported testing positive.

### Adherence to COVID-19 public health guidelines

**Fig. 1:**
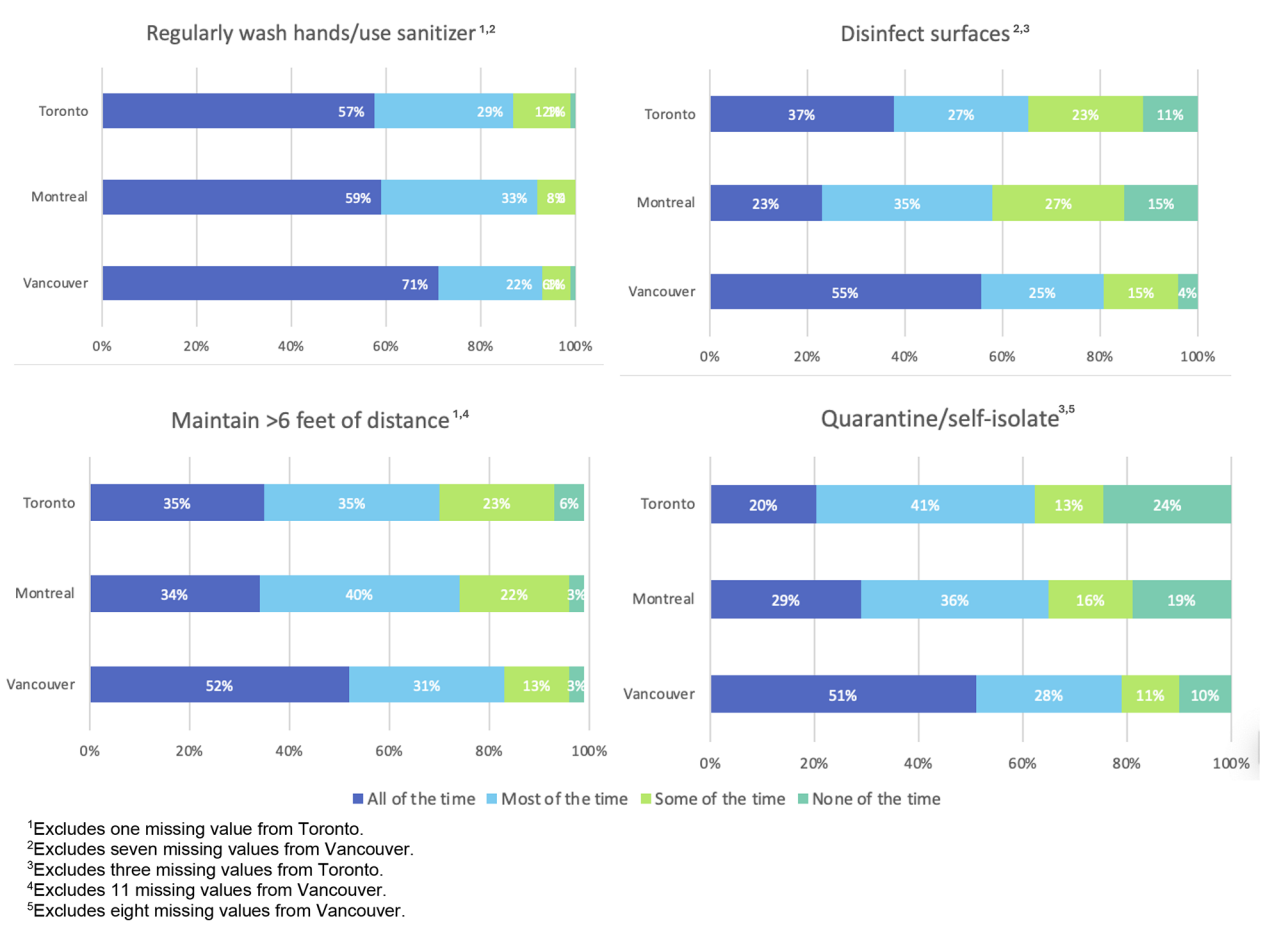
Adherence to COVID-19 public health guidelines among sampled people who use drugs across three Canadian cities, May 2020–March 2021 (*N* = 1,025). *Notes:* All percentages are calculated with missing responses included in the denominator

A majority of participants at all three sites reported adhering to protective measures including regularly washing their hands or using hand sanitizer, disinfecting surfaces, maintaining physical distancing, and self-isolating, all or most of the time (Fig. 1). However, 24% and 19% of participants in Toronto and Montreal reported being able to self-isolate none of the time.

### Impacts of COVID-19 on meeting essential needs

Overall, 15% of Vancouver participants, 44% of Toronto participants, and 32% of Montreal participants reported a change in their living situation during the pandemic. However, this was not always a negative change; while 33–36% of participants in each site reported that their living situation had worsened, between 43 and 49% reported an improvement. With respect to reasons for a change in living situation, a greater proportion of participants in Vancouver (22%) and Toronto (36%) compared to Montreal (7%) reported receiving new shelter or housing. Compared to their counterparts in Vancouver and Toronto, participants in Montreal more often also attributed their change in living situation to moving in order to self-isolate (14%), being asked to leave by others (12%), and no longer being able to afford rent (10%). One in five Montreal participants and one in ten Toronto participants reported becoming homeless because of the health emergency; however, this phenomenon was less common among participants in Vancouver (Table 2).

Going hungry more often since the start of the pandemic was reported by 20% of participants in Vancouver, 41% in Toronto, and 38% in Montreal. Additionally, 40% and 36% of participants in Toronto and Montreal and 19% of participants in Vancouver reported a decrease in monthly income since the start of the COVID-19 pandemic. However, during the same period, 48% of participants in Vancouver, 39% in Toronto, and 17% in Montreal reported an increase in monthly income.

### Impacts of COVID-19 on access to harm reduction and drug treatment services

As shown in Tables 3 and 35%-56% of sampled participants reported receiving opioid agonist therapy (OAT) in the past six months. Of these participants, most reported continuing to receive their treatment during the COVID-19 health crisis (87-93%). However, among those receiving OAT at the time of the interview, 32% and 22% of participants in Toronto and Montreal reported missing doses due to service disruptions (not reported in Vancouver). Despite these disruptions, across all three sites, very few participants discontinued OAT during the COVID-19 health crisis, and fewer still discontinued OAT for reasons related to the pandemic.

**Table 2:** Impacts of the COVID-19 pandemic on essential needs among sampled people who use drugs across three Canadian cities, May 2020–March 2021 (*N* = 1,025)

	Vancouver (*N* = 640)	Toronto (*N* = 158)	Montreal (*N* = 227)
**Impact on living situation**			
**Change in living situation**^**1**^, **n (%)** Yes No	95 (15) 542 (85)	69 (44) 89 (56)	73 (32) 154 (68)
**Quality of new living situation**^**2,3**^, **n (%)** Better Worse About the same Don’t know	45 (49) 31 (33) 16 (17) 0 (0)	30 (43) 24 (35) 11 (16) 3 (4)	34 (47) 26 (36) 22 (15) 2 (1)
**Reason for new living situation**^**2,4,5**^, **n (%)** Moved to be with family/partner Moved to be away from a vulnerable family member Moved to self-isolate Asked to leave by others Received new shelter or housing Could no longer afford rent Other	8 (9) 1 (1) 3 (3) 4 (4) 20 (22) 2 (2) 58 (62)	3 (4) 1 (1) 2 (3) 2 (3) 25 (36) 0 (0) 41 (59)	4 (5) 1 (1) 10 (14) 9 (12) 5 (7) 7 (10) 38 (52)
**Impact on survival-related needs**			
**Became homeless**^**6**^, **n (%)** Yes No	16 (3) 622 (97)	16 (10) 141 (89)	42 (19) 185 (81)
**Change in going hungry**^**7**^, **n (%)** Yes, more often than usual Yes, less than usual No, same as usual	125 (20) 30 (5) 481 (76)	64 (41) 25 (16) 60 (38)	85 (38) 29 (13) 112 (49)
**Change in monthly income**^**8**^, **n (%)** Yes, income has decreased Yes, income has increased No, income has not changed	122 (19) 308 (48) 207 (33)	63 (40) 61 (39) 31 (20)	82 (36) 38 (17) 105 (46)

*Notes*: All percentages are calculated with missing responses included in the denominator.

^1^Excludes 3 missing values from Vancouver.

^2^Calculated among participants who reported experiencing a change in living situation since the COVID-19 emergency was declared.

^3^Excludes one refusal in Vancouver and one missing value in Toronto.

^4^Excludes one “don’t know” in Montreal, and two “don’t know” and one refusal in Vancouver.

^5^Participants could select multiple responses.

^6^Excludes one missing value from Toronto.

^7^Excludes eight missing values and one “don’t know” response from Toronto, and one missing value from Montreal.

^8^Excludes two “don’t know” and one missing value from Montreal, and one “don’t know”, one refusal, one “don’t know”, and one missing value from Toronto.

**Table 3:** Impacts of the COVID-19 pandemic on access to drug treatment and harm reduction services among sampled people who use drugs across three Canadian cities, May 2020–March 2021 (*N* = 1,025)

	Vancouver (*N* = 640)	Toronto (*N* = 158)	Montreal (*N* = 227)
**Impact on continuity of opioid agonist therapy**			
Received OAT in the past six months, n (%)	357 (56)	83 (53)	79 (35)
Currently receiving OAT^1,2^, n (%)	330 (93)	72 (89)	69 (87)
Missed doses due to service disruptions^3^, n (%)	NR	23 (32)	15 (22)
Left OAT during the COVID-19 health crisis^2^, n (%)	12 (3)	9 (11)	10 (13)
Left for reasons related to the COVID-19 health crisis^4^, n (%)	1 (8)	2 (22)	1 (10)
**Impact on access to harm reduction services**			
**Attempted to access the following services, but unable to**^**5**^, **n (%)** Supervised consumption site Drug checking service Sterile needle-syringes Naloxone	76 (38) 45 (44) NA 24 (9)	72 (52) 5 (12) 28 (20) 9 (10)	12 (26) 1 (5) 5 (6) 2 (5)

*Notes*: All percentages are calculated with missing responses included in the denominator.

NA = not applicable (question not asked at site); NR = not reported; OAT = opioid agonist therapy.

^1^Excludes one missing value from Toronto.

^2^Percentage calculated among those on OAT in the past six months.

^3^Percentage calculated among those currently on OAT; excludes two missing values from Toronto.

^4^Percentage calculated among those who were on OAT in the past six months and left OAT during the COVID-19 health crisis.

^5^Percentages are calculated among those who attempted to access the service. Supervised consumption sites: *n* = 47 in Montreal, *n* = 138 in Toronto, *n* = 199 in Vancouver. Sterile needle-syringes: *n* = 87 in Montreal, *n* = 141 in Toronto. Naloxone: *n* = 37 in Montreal, *n* = 90 in Toronto, *n* = 275 in Vancouver. Drug checking service: *n* = 24 in Montreal, *n* = 41 in Toronto, *n* = 102 in Vancouver.

Attempts to access harm reductions services during the pandemic varied across cities and services: 20-87% for supervised consumption sites, 38-89% for sterile syringes (data not available in Vancouver), 16-57% for naloxone, and 11-16% for drug checking services. Among participants trying to access harm reduction services, some reported difficulty in doing so; in particular supervised consumption sites across all three cities (ranging from 26% in Montreal to 52% in Toronto), and drug checking services in Vancouver (44% of participants). Fewer participants across sites reported difficulty accessing naloxone or sterile injecting equipment.

### Impacts of COVID-19 on overdose risk, drug use behaviours, and experiences of violence

Overall, 102 Vancouver participants (16%), 52 Toronto participants (33%), and 19 Montreal participants (8%) reported experiencing a non-fatal overdose in the past six months during the COVID-19 pandemic. However, across all three sites, the majority of participants reported no change in their frequency of experiencing overdose (67–91%).

A majority of participants in all three cities generally did not report changes to their overall use of injection or non-injection drugs, their frequency of injecting while alone, or their frequency of injecting in public (Table 4). However, we did detect city-specific variations: 36% of participants in Montreal reported an increase in non-injection drug use; 22% and 20% of participants in Vancouver reported an increase in non-injection and injection drug use, while 26% of participants in Toronto reported an increase in injection drug use. A greater proportion of participants in Toronto further reported increases in injecting in public and alone (18% and 27%) compared to their counterparts in Vancouver and Montreal (injecting in public: 5% and 6%; injecting alone: 10% and 5%).

Participants reported mixed experiences of violence since the COVID-19 health emergency was declared. Across all sites, most participants reported little change in the frequency of violence they witnessed being directed toward other people (41-46%), physical or sexual assault directed toward themselves (physical assault: 50-87%; sexual assault: 59-97%), feelings of being threatened or unsafe (53-59%), or violence perpetrated by the police (43-60%; Table 4). Slightly less than a third of participants in Toronto and Montreal, however, reported more frequent experiences of physical violence and feeling threatened during the pandemic, and about 40% of participants from each of these settings reported witnessing violence against other people more often.

**Table 4:** Impacts of the COVID-19 pandemic on drug use behaviours and experiences of violence among people who use drugs across three Canadian cities, May 2020–March 2021 (*N* = 1,025)

	Vancouver (*N* = 640)	Toronto (*N* = 158)	Montreal (*N* = 227)
**Impact on drug use patterns and behaviours**			
**Overall use of non-injection drugs**^**1**^, **n (%)** Yes, has increased Yes, has decreased No, has not changed	111 (22) 87 (17) 318 (62)	27 (17) 25 (16) 103 (65)	81 (36) 52 (23) 94 (41)
**Overall use of injection drugs**^**2**^, **n (%)** Yes, has increased Yes, has decreased No, has not changed	81 (20) 97 (23) 233 (57)	41 (26) 47 (30) 67 (42)	36 (16) 32 (14) 158 (70)
**Frequency of injecting drugs alone**^**3**^, **n (%)** Yes, frequency has increased Yes, frequency has decreased No, frequency has not changed	35 (10) 16 (4) 308 (85)	42 (27) 28 (18) 83 (53)	12 (5) 15 (7) 67 (87)
**Frequency of injecting drugs in public**^**4**^, **n (%)** Yes, frequency has increased Yes, frequency has decreased No, frequency has not changed	19 (5) 20 (6) 317 (88)	29 (18) 45 (28) 79 (50)	14 (6) 13 (6) 65 (86)
**Impact on experiences of violence**			
**Witnessed violence against other people**^**5**^, **n (%)** More than usual About the same as usual Less than usual Don’t know/refused	NA NA NA NA	59 (37) 73 (46) 20 (13) 5 (3)	93 (41) 94 (41) 19 (8) 21 (9)
**Experienced physical violence**^**5**^, **n (%)** More than usual About the same as usual Less than usual Don’t know/refused	55 (9) 550 (87) 18 (3) 6 (1)	43 (27) 79 (50) 33 (21) 2 (1)	66 (29) 122 (54) 14 (6) 25 (11)
**Experienced sexual assault**^**6**^, **n (%)** More than usual About the same as usual Less than usual Don’t know/ refused	10 (2) 611 (97) 3 (1) 5 (1)	10 (6) 113 (72) 30 (19) 3 (2)	12 (5) 133 (59) 6 (3) 76 (33)
**Felt threatened or unsafe**^**5**^, **n (%)** More than usual About the same as usual Less than usual Don’t know/refused	NA NA NA NA	51 (32) 83 (53) 21 (13) 2 (1)	65 (29) 134 (59) 10 (4) 17 (7)
**Experienced police violence**^**5,7**^, **n (%)** More than usual About the same as usual Less than usual Don’t know/refused	11 (38) 13 (45) 5 (17) 0	34 (22) 68 (43) 53 (34) 2 (1)	48 (21) 137 (60) 14 (6) 27 (12)

*Notes*: All percentages are calculated with missing responses included in the denominator.

NA = not applicable (question not asked at site).

^1^Excludes one “don’t know”, and two missing values from Toronto.

^2^Excludes one “don’t know” in Vancouver, and one “don’t know” and two missing values from Toronto.

^3^Excludes excludes one “don’t know” and one refusal in Vancouver, and two “don’t know”, one refusal, and two missing values from Toronto.

^4^Excludes one “don’t know” and three refusals from Vancouver, and two “don’t know”, one refusal, and two missing values from Toronto.

^5^Excludes one missing value from Toronto.

^6^Excludes two missing values from Toronto.

^7^Question was asked to a subset of participants from Vancouver who were stopped, detained, or searched by police.

## Discussion

The initial phases of the COVID-19 pandemic worsened existing social and health inequities, and introduced additional barriers to meeting essential needs and accessing services among people who use drugs living in Canada’s three largest cities. These challenges included service access barriers, as well as heightened experiences of loss of income, food insecurity, housing instability, exposure to violence, and police interactions. Challenges experienced varied across sites, with some indication that participants from Vancouver fared somewhat better compared to their counterparts in Toronto and Montreal with respect to income and food security, changes in their living situation, and continuity of OAT. Nonetheless, participants across sites largely reported adhering to public health directives to prevent SARS-CoV-2 transmission, and the self-reported prevalence of SARS-CoV-2 infection was low.

These results have important implications for efforts to improve preparedness measures in anticipation of future public health emergencies such as pandemics. First, these findings suggest that for some people who use drugs, the COVID-19 pandemic amplified existing health and social inequities, although this experience was not uniform [16, 23, 24]. In Vancouver, Toronto, and Montreal, 48%, 39%, and 19% of participants reported increases in income. Although the source of the income increase was not captured in our data, our findings suggest that it is possible that federal government efforts to provide financial support via the Canada Emergency Response Benefit [42] were accessed by some individuals shouldering economic burden. Equally important, participants across settings also reported decreases in income and greater food insecurity, with individuals in Vancouver experiencing better outcomes in these indicators compared to participants in Toronto and Montreal. These findings are corroborated by other investigations among people who use drugs which found that pandemic-related restrictions negatively impacted income generation and ability to meet material needs, primarily due to job loss and decreased ability to engage in informal street-based income generation activities (i.e., panhandling) [15, 24]. We also found that across sites, among participants who experienced a change in living situation, slightly less than half reported improvements while over a third reported that their living circumstances worsened. Furthermore, approximately 1 in 5 participants in Montreal and 1 in 10 participants in Toronto reported becoming homeless as a result of the pandemic. Although findings should be considered in light of baseline measures of housing instability in these settings prior to the pandemic, they nonetheless indicate high—and increasing—unmet housing needs in this population during this time. While housing instability is common among people who use drugs and may in part explain reported changes in housing circumstances among participants [43], many Canadian cities implemented temporary shelters and hotel programs to accommodate people experiencing homelessness in the initial phase of COVID-19 restrictions so that they could comply with public health distancing ordinances [15, 16]. These temporary housing programs were not uniformly implemented across cities, and permanent housing shelters either closed or underwent critical operational changes, negatively impacting equity-seeking groups, including people who use drugs [16]. Given moderately high levels of homelessness experienced by participants in Montreal and Toronto during the pandemic, preparedness measures must prioritize equitable access to stable, safe, and affordable housing in advance of public health emergencies or other large-scale disruptive events [15]. Interventions to consider include rent or mortgage deferrals, anti-eviction policies, and sustainable options for temporary and transitional housing [15].

Although settings across the United States and Canada have witnessed sharp increases in drug poisoning deaths since the onset of the pandemic which have been attributed to COVID-19 infection control measures and related service access restrictions [15, 21, 44], we found that a majority of participants across all three cities reported no change in their frequency of experiencing non-fatal overdose during the pandemic. This may be explained by several reasons. Self-reported non-fatal overdose may have subjected findings to survival bias, unable to capture those who have died from fatal overdose. It is further possible that some study participants may have already been highly susceptible to drug poisoning risk, resulting in little change in their perception of overdose experiences during the COVID-19 pandemic. Although participants perceived little change in their experience of overdose, aforementioned disruptions within socioeconomic risk environments attributed to COVID-19 restrictions are consistent with city-wide drug poisoning morality data [45].

Approximately half of participants across each site reported concerns related directly to the COVID-19 pandemic. Combined with increased socioeconomic precarity and disruption in access to essential services, such stressors may have been in part responsible for the observed increase in overall drug use among some participants as a means to cope or self-medicate. Other qualitative studies have documented similar reasons for increases in drug use among people who use drugs during the pandemic including concerns related financial uncertainty, fear of catching COVID-19, and boredom associated with job loss and isolation from one’s family and friends [14, 15, 21, 25]. In Toronto and Montreal, we further observed that approximately one-third to two-fifths of participants reported experiencing physical violence, witnessing violence, and feeling threatened or unsafe more often than usual during the pandemic. While people who use drugs have been known to disproportionately experience with high rates of violence prior to the pandemic, it is possible that pandemic-related restrictions may have led to the further intensification of violence experienced by this population for various reasons, including increased exposure to violent relationships resulting from physical distancing directives, disruptions in access to critical services that offer refuge or support, heightened uncertainty and stress contributing to increased perpetration of violence, as well as the possible impact of supply interruptions within local drug markets [46].

The findings presented here indicated high levels OAT treatment continuity, consistent with other analyses of administrative health data linked to the OiSIS-Toronto cohort and Ontario-wide registries which found high levels of sustained retention of individuals enrolled in OAT during the COVID-19 pandemic [47, 48]. This suggests that efforts to increase OAT prescribing and retention, such as ‘temporary exemptions’ for the prescription of controlled medications, national guidance for prescribers and pharmacists with patients on OAT, and measures to increase program flexibility such as increased take-home ‘carries’ and decreased requirements for urine screens [49, 50], were generally effective. Nevertheless, in Montreal and Toronto, approximately one-fifth and one-third of participants who were enrolled in OAT at the time of the interview missed doses due to service disruptions, and 13%, 11%, and 3% of participants in Montreal, Toronto, and Vancouver discontinued OAT during the COVID-19 health crisis, suggesting that the aforementioned efforts did not meet the treatment needs for all. This is consistent with a national qualitative study of people who use drugs across Canada, wherein a large portion of OAT users reported negative pandemic-related changes to OAT provision, including missed doses and treatment discontinuation [16]. Collectively this research highlights the importance of evaluating ongoing reforms to OAT policy in order to mitigate gaps in substance use treatment continuity during public health emergencies [44].

Finally, the experiences of accessing harm reduction services during the pandemic were mixed among participants. Of participants who sought to obtain naloxone and sterile syringes, few reported difficulties in doing so. However, between a quarter to a half of participants across all three sites reported major challenges accessing supervised consumption sites, which aligns with previous research on the Canadian pandemic context [16, 21, 22, 51]. Reasons for limited access to these services cited by other work include the closure of sites, reduced hours of operations, staff shortages, and reduced physical capacity of services, which subsequently led to longer wait times [16, 21, 22]. Given the well-established impact of these sites in reducing deaths [52, 53], and the increase in population-level incidence of drug poisoning mortality in many provinces during the COVID-19 pandemic [54], efforts to maintain the operation of supervised consumption sites should be prioritized during and beyond the COVID-19 pandemic era.

In an effort to ensure that future preparedness measures are equitable and responsive, we highlight a range of pandemic-related service gaps experienced by people who use drugs during the COVID-19 pandemic, when drug poisoning mortality increased precipitously across Canada. While we observed trends that were broadly consistent across sites, our findings indicated that COVID-19-related impacts were not uniform, with greater percentages of participants in Vancouver experiencing better outcomes related to income and food security, housing status, and OAT continuity compared to participants in Toronto and Montreal. Although reasons for these variations remain unexplored, Vancouver’s Downtown Eastside neighbourhood–the primary recruitment site for the Vancouver cohorts–differs from other settings, characterized by its longstanding history of drug user advocacy and its concentrated infrastructure of harm reduction and treatment services prior to the pandemic [55–57]. Such variations point to future areas of investigation that take into consideration cross-jurisdictional comparisons of socioeconomic risk environments, and health and social service and policy arrangements for optimal pandemic and emergency-related responses.

In sum, to prevent the inadvertent increased risk of death and illness associated with drug poisonings and other drug-related outcomes, future risk mitigation strategies in response to public health emergencies must take into account the basic needs of people who use drugs. Failing to do so would further amplify enduring health inequities experienced by equity-seeking populations and thereby reduce the likelihood of achieving global public health priorities as articulated by the UN Research Roadmap for the COVID-19 Recovery [58]. To maintain continuity of essential care, harm reduction and treatment services should prioritize the creation of up-to-date emergency preparedness response protocols for a broad range of emergencies, with particular emphasis on reaching those facing intensified socioeconomic hardship (i.e., unemployment, insufficient income, housing precarity) [8]. Other related strategies may involve establishing temporary outdoor or mobile harm reduction services, integrating digital health technologies that provide care through telecare services, or allowing for the remote monitoring of drug use to intervene in case of emergencies [59]. These approaches may also have the secondary benefit of expanding harm reduction services and supervised consumption sites to rural, remote, and suburban communities that do not currently have access, and which are disproportionately impacted by drug poisoning [60]. Finally, ensuring expanded access to low-barrier health and social services (e.g., food bank services, income supports) and optimizing flexible OAT protocols to account for pandemic-related restrictions should remain an ongoing priority.

This study has a number of limitations. First, cross-sectional data were generated from subsamples of observational cohorts of people who use drugs, which employed distinct convenience sample-based approaches for recruitment. As such, results are not representative of the wider population of people who use drugs in these settings, and elsewhere. Given that a sampling frame or registries of people who use drugs do not exist, this limitation is typical of all cohorts of people who use drugs [61, 62]. Given that this work was undertaken in large urban municipalities, study findings may also not be reflective of people who use drugs from rural and remote Canadian settings where drug supply and health service and harm reduction policy and program arrangements may differ. Second, the differences in recruitment methods, inclusion criteria, and data collection strategies precluded pooling data or conducting cross-site analyses or formal comparisons. Third, given COVID-19 restrictions on in-person interviews, participants were contacted and interviewed by phone or email; and as such, the sample may reflect those facing less socioeconomic barriers. This may also reflect individuals whose lives were less disrupted by pandemic restrictions and were thus able to connect with study staff. Fourth, data across sites were collected between May 2020 and March 2021, and therefore are only indicative of those interviewed during this period. Fifth, given that the Toronto-based cohort was established more recently (i.e., November 2018) and specifically to assess the impact of supervised consumption sites on health and social outcomes among people who use drugs, participants in this setting may have been more likely to access these sites and also more difficult to maintain contact with once sites were closed or had their hours reduced. Finally, slightly over 40% of Montreal participants were drawn from a convenience-based sample of people who use drugs recruited from low-threshold community-based programs, possibly capturing a subset of individuals facing increased marginalization. In contrast to participants in Toronto and Montreal, participants in Vancouver were exclusively drawn from longstanding community-recruited cohorts.

## Conclusions

The COVID-19 pandemic impacted the health and well-being of people who use drugs across three Canadian cities in multiple and divergent ways. The varied experiences of service access, continuity of care, and economic security presented herein—along with an increase in the population-level incidence of drug poisoning mortality across Canada—suggest that, by and large, the needs of people who use drugs were not adequately met during this time. Future equitable preparedness efforts that respond to pandemics and other emergencies must proactively recognize and mitigate the amplification of health and social inequities that were experienced during the COVID-19 pandemic, with special consideration of jurisdictional context. The failure to do so could exacerbate health-related risks experienced by people who use drugs facing the ongoing threat of drug poisoning in Canada, the United States, and internationally.

## Data Availability

Public sharing of data for this study is not permitted under the parameters of the research ethics approval, given potentially identifying and sensitive health and legal information. For further inquiries, please contact the corresponding author at dwerb@health.ucsd.edu.
